# Editorial to Summarize the Papers Published in the Special Issue “10th Anniversary of Cells—Advances in Cell Cycle”

**DOI:** 10.3390/cells11152437

**Published:** 2022-08-05

**Authors:** Zhixiang Wang

**Affiliations:** Signal Transduction Research Group, Department of Medical Genetics, Faculty of Medicine and Dentistry, University of Alberta, Edmonton, AB T6G 2H7, Canada; zhixiang.wang@ualberta.ca

To celebrate its 10th anniversary, the prestigious journal *Cells* launched a series of Special Issues in 2021. This Special Issue entitled “10th Anniversary of Cells—Advances in Cell Cycle” was launched together with other sister Special Issues under the umbrella “10th Anniversary of Cells”. I am honored to be invited to serve as the academic editor for this Special Issue. This Special Issue attracted the attention of many scientists in the cell cycle field and consists of 10 high quality papers, including 4 research articles and 6 scientific reviews—a great success. The four research articles focus on various important topics of the cell cycle using a broad range of model organisms, including yeast, sea urchins, green algae, and human cancer cell lines.

The first research article published in this issue is focused on the cell cycle progression of green algae *Desmodesmus quadricauda* [1]. The cell cycle progression of D. quadricauda consists of cell growth, duplication, and division, which is usually examined by a biochemical analysis of macromolecules (RNA, protein, DNA, and starch). However, such analyses are rather time consuming, complicated, and require a large quantity of initial samples. Czech scientist Bišová and her team aimed to find a more efficient way to analyze these large biomolecules. In their research, they used two independent methodologies to examine the dynamics of starch, lipid, polyphosphate, and guanine pools during the cell cycle of the synchronized green algae: a conventional biochemical analysis of cell suspensions and confocal Raman microscopy of single cells. They concluded that confocal Raman microscopy can detect even low levels of macromolecules naturally present in the cells during their vegetative development. Confocal Raman microscopy is especially suited for the detection of polyP, lipids, and guanine crystals within cells [1].

Mitosis is the most dynamic period of the cell cycle, involving a major reorganization of virtually all the cell components. Mitosis is divided into prophase, prometaphase, metaphase, anaphase, and telophase. The progression of the cell cycle through these mitotic subphases is tightly regulated by complicated molecular mechanisms. Mitosis is also the most fragile period of the cell cycle. Therefore, most cancer drugs are designed to specifically target mitotic cells. The next two research articles published in this Special Issue focused on the mitotic phase of the cell cycle [2,3].

The research article by Bari et al., aimed to develop a novel therapeutic agent against Glioblastoma multiforme (GBM) by arresting the cancer cells in mitosis [2]. GBM is the most malignant and frequent human brain tumor, representing more than 60% of all brain tumors in adults [2]. Despite the great advances made in understanding the molecular alterations that occur in Glioblastoma (GB), there is no definitive cure, and mortality is still very high. Therefore, the development of new therapeutic agents is clinically urgent. The Italian research team previously led by Dr. Tata showed that the arecaidine propargyl ester (APE), an orthosteric agonist of M2 muscarinic acetylcholine receptors (mAChRs), arrests the cell cycle of glioblastoma (GB) cells and reduces their survival. They showed that M2 agonist treatment causes an arrest in cell cycle progression with an accumulation of cells during the pro-metaphase/metaphase transition, which causes a significant increase in abnormal mitosis and multipolar mitotic spindle formation [2]. These findings highlight the M2 muscarinic receptor as a new strategic therapeutic target in GBM therapy.

Vannini et al., studied the regulation of the mitotic exit in the budding yeast *Saccharomyces cerevisiae* [3]. The mitotic exit is critical for the successful completion of a cell division cycle. The mitotic exit network (MEN) is a Ras-like signal transduction pathway that promotes mitotic exit in anaphase. A crucial step in MEN activation is the activation of Cdc15, which requires the association of Cdc15 with spindle pole bodies (SPBs) and the presence of the Tem1 GTPase and the Polo kinase Cdc5. However, it was previously unclear how Cdc15 associated with SPBs. In this research, a collaborative team led by Dr. Seshan identified a hyperactive allele of NUD1, nud1-A308T, that recruits Cdc15 to SPBs in all stages of the cell cycle in a CDC5-independent manner. They further showed that nud1-A308T leads to the early recruitment of Dbf2-Mob1 during metaphase [3]. Their findings highlight the importance of scaffold regulation in signaling pathways to prevent improper activation.

In the fourth research article, Limatola et al., studied the role of the vitelline layer (VL) of sea urchin (*Lytechinus pictus*) eggs in fertilization [4]. Sea urchins have been a model organism in cell cycle research and contributed to the discovery of cyclins [5]. In this study, the research team led by Italian scientist Santella attempted to partially disrupt the VL with a reducing agent, dithiothreitol (DTT), and then observed its effects on fertilization. They showed that DTT treatment did not elevate the fertilization envelop, but instead caused a few anomalies at fertilization, including compromised Ca2+ signaling, the blocked translocation of cortical actin filaments, and impaired cleavage. The authors concluded that the fertilization envelop is not the decisive factor preventing polyspermy, and that the integrity of the VL is crucial to the egg’s fertilization response [4].

This Special Issue also published six comprehensive high quality review articles. These reviews covered a broad range of topics: the impact of 5-Bromo-2′-deoxyuridine (BrdU) on the proliferative behavior of cerebellar neuroblasts [6], the regulation of the cell cycle by telomerase [7], growth factors [8], heat shock transcription factors [9], Cyclin-Dependent Kinases and CTD Phosphatases [10], and the effects of low-dose radiation on the cell cycle [11].These reviews focused on various model organisms, including mammalian cells, plant cells, yeast, and parasites of the *Leishmania genus*.

As a marker for DNA synthesis, BrdU has generated important insights into the cellular mechanisms underlying the proliferative behavior of neuroblasts. BrdU labeling is the most widely used procedure for studying cell-cycle phases and their durations, as well as to identify dividing neuroblasts and follow their fates. Even though BrdU has demonstrated toxicity, its detrimental effects on the proliferation and viability of different cell types have been frequently neglected. In a focused review article that included numerous data from his own lab, Dr. Martí-Clúa evaluated the potential negative impact of using BrdU to study the proliferation of neuroblasts [6]. He found that incorporation of BrdU into newly synthesized DNA may lead to inaccurate results. Thus, caution should be exercised when interpreting the results obtained using BrdU. This is particularly important when high or repeated doses of this agent are used.

Leishmaniasis are a group of common poverty-related endemic diseases. They cause a wide spectrum of clinical manifestations, and approximately one million new diagnoses are expected yearly in East Africa, the Indian subcontinent, and Latin America. Thus, novel treatments against leishmaniasis are urgently needed. For this purpose, molecular biology studies on Leishmania spp. have been conducted to elucidate the different aspects of parasite biology. Among these studies, trypanosomatid telomere biology has generated great interest in the scientific community, as telomeres are essential for genome stability and cell cycle progression. In this comprehensive review, Dr. Cano and her colleagues aim to shed light on what we know about the phenomena behind telomere maintenance and how it impacts the parasites’ cell cycle and survival [7]. Their review covers the knowledge available so far on the Leishmania spp. cell cycle and telomere homeostasis, with an emphasis on the remaining gaps and the advances reached in the last few years. Among the impressive progress made on understanding the biology of these parasites are the facts that they remain in the G0 state during their infective stages and the remarkable divergence of their telomeric shelterin-like complex relative to mammals. However, these aspects are only a few examples of how this subject can be linked in the future to leishmaniases treatment and how far scientists are from a deeper knowledge of these peculiar eukaryote parasites.

A review I authored myself focuses on the regulation of cell cycle progression by growth factor (GF)-induced cell signaling [8]. The driving force of cell cycle progression are GF-initiated signaling pathways that control the activity of various Cdk-cyclin complexes. While the mechanism underlying the role of GF signaling in the G1 phase of the cell cycle progression is well understood, little is known regarding the function and mechanism of GF signaling in regulating other phases of the cell cycle, including the S, G2, and M phases. In this review, we briefly discuss the history of cell cycle research and the process of cell cycle progression through various phases. The emphasis in this research is on the role of signaling pathways activated by GFs and their receptors (mostly receptor tyrosine kinases) in regulating cell cycle progression through various phases. As summarized in the review, the accumulated results so far suggest that GF signaling may regulate cell cycle progression throughout all phases of the cell cycle, but further research is needed to sustain these findings and to uncover the underlying molecular mechanisms [8].

Heat shock transcription factors (HSFs) have been noted as critical proteins for cells’ survival against various stresses; however, recent studies suggest that HSFs also have important roles in cell cycle regulation-independent cell-protective functions. During the cell cycle progression, HSF1 and HSF2 bind to condensed chromatin to provide immediate precise gene expression after cell division. In a comprehensive review, Dr. Hayashida and his colleagues discussed the function of these HSFs in cell cycle progression, cell cycle arrest, gene bookmarking, mitosis, and meiosis [9]. They briefly described the early essential findings in cell cycle studies and the discovery of the heat shock response and the essential functions of HSFs. They further describe all of the important discoveries to date regarding the function of HSFs in cell cycle regulation, including the interaction between HSF1 and p53 as well as HSFs and WD40 repeat proteins [9].

The precise control of transcription is crucial for the synthesis of many phase-specific proteins and for the orderly progression of the cell cycle. Dr. Zheng wrote a timely review for this Special Issue to discuss this important topic in cell cycle research [10]. This comprehensive review highlights highly conserved transcriptional regulators that are shared in budding yeast (Saccharomyces cerevisiae), Arabidopsis thaliana model plants, and humans, which are separated by more than a billion years of evolution. In addition, the functions of plant-unique regulators in relation to cell division are also discussed. The regulators discussed in the review include structurally and/or functionally conserved regulators such as cyclin-dependent kinases (CDKs) and RNA polymerase II C-terminal domain (CTD) phosphatases. The nature of the classical versus the shortcut models of Pol II transcriptional control is also discussed. These CDKs and CTD phosphatases have conserved domains across the three eukaryotic kingdoms, but some of them have also evolved with unique structures and functions in each of the kingdoms. This review is an insightful future perspective regarding the precise control of transcription in cell cycle regulation [10].

This Special Issue is concluded by a well-written comprehensive review regarding the effects of low-dose radiation on the cell cycle [11]. Cells exposed to ionizing radiation undergo a series of complex responses, including DNA damage, reproductive cell death, and altered proliferation states, which are all linked to cell cycle dynamics. Ionizing radiation has been used in cancer treatment for more than one hundred years and is currently a standard option in treating 20–60% of all new cancer cases. Through the years, extensive research has been conducted on cell cycle checkpoints and their regulators in mammalian cells in response to high-dose ionizing radiation. However, it is unclear how low-dose ionizing radiation (LDIR) regulates the cell cycle progression. In this review, Drs. Khan and Wang aim at summarizing the recent advances related to the role of LDIR (200 mGy or lower) in cell cycle regulation. This review also discusses how different cell cycle regulators are modulated by LDIR exposure in normal, cancerous, and stem cells. Moreover, the authors propose a new conceptual mechanism to explain how LDIR differentially regulates the nucleocytoplasmic shuttling of p21^Waf1^, a key cell cycle regulator and transcriptional target of p53 [11].

Cell cycle control is vital for cell proliferation in all eukaryotic organisms. Through more than one hundred years of efforts by scientists, we now have a much clearer picture of cell cycle progression and its regulation. The research articles and reviews published in this Special Issue reflect the current and future efforts made by scientists to better understand the progression and regulation of the cell cycle, and how they may serve as targets for novel cancer therapies.
